# Supplementary material for: Tropical deforestation causes large reductions in observed precipitation
Source: Nature. 2023 Mar 1;615(7951):270–5. doi: 10.1038/s41586-022-05690-1 (PMC9995269; doi:10.1038/s41586-022-05690-1)
Supplement: Supplementary file 1 — This file Supplementary Figs. 1–10, which support the results presented in the main paper. Here we also present results from the individual precipitation datasets. [file 41586_2022_5690_MOESM1_ESM.pdf]

nature portfolio

https://doi.org/10.1038/s41586-022-05690-1

Supplementary information

Tropical deforestation causes large reductions in observed precipitation

In the format provided by the authors and unedited

Supplementary Information for paper:
"Tropical deforestation causes large reductions in observed precipitation"

Figures

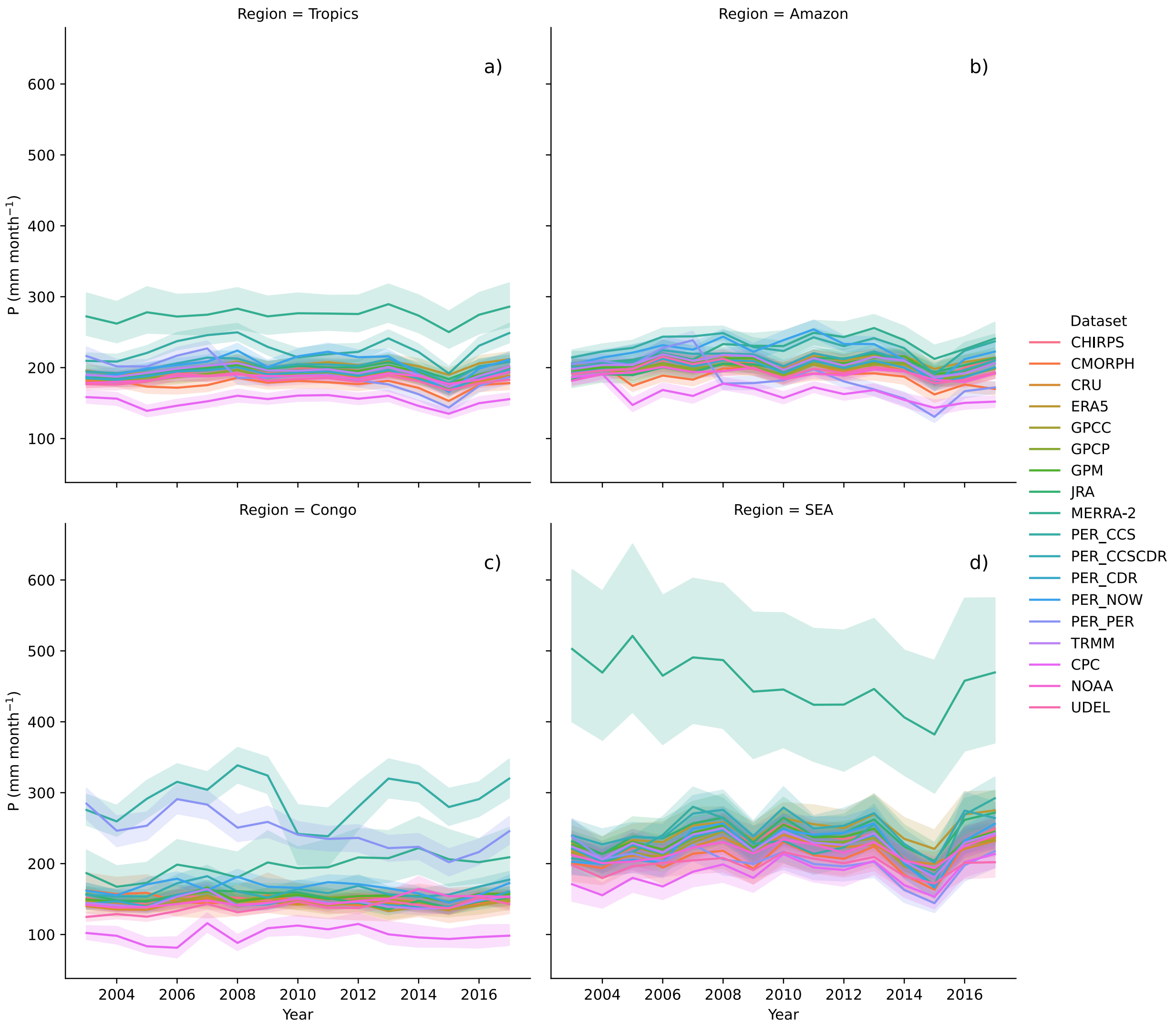

Supplementary Figure 1. Mean annual precipitation (P, mm month^{-1}). a) Tropics, b) Amazon, c) Congo and d) SEA. Details of each data product are provided in Extended Data Table 1. Shading indicates 95% confidence intervals.

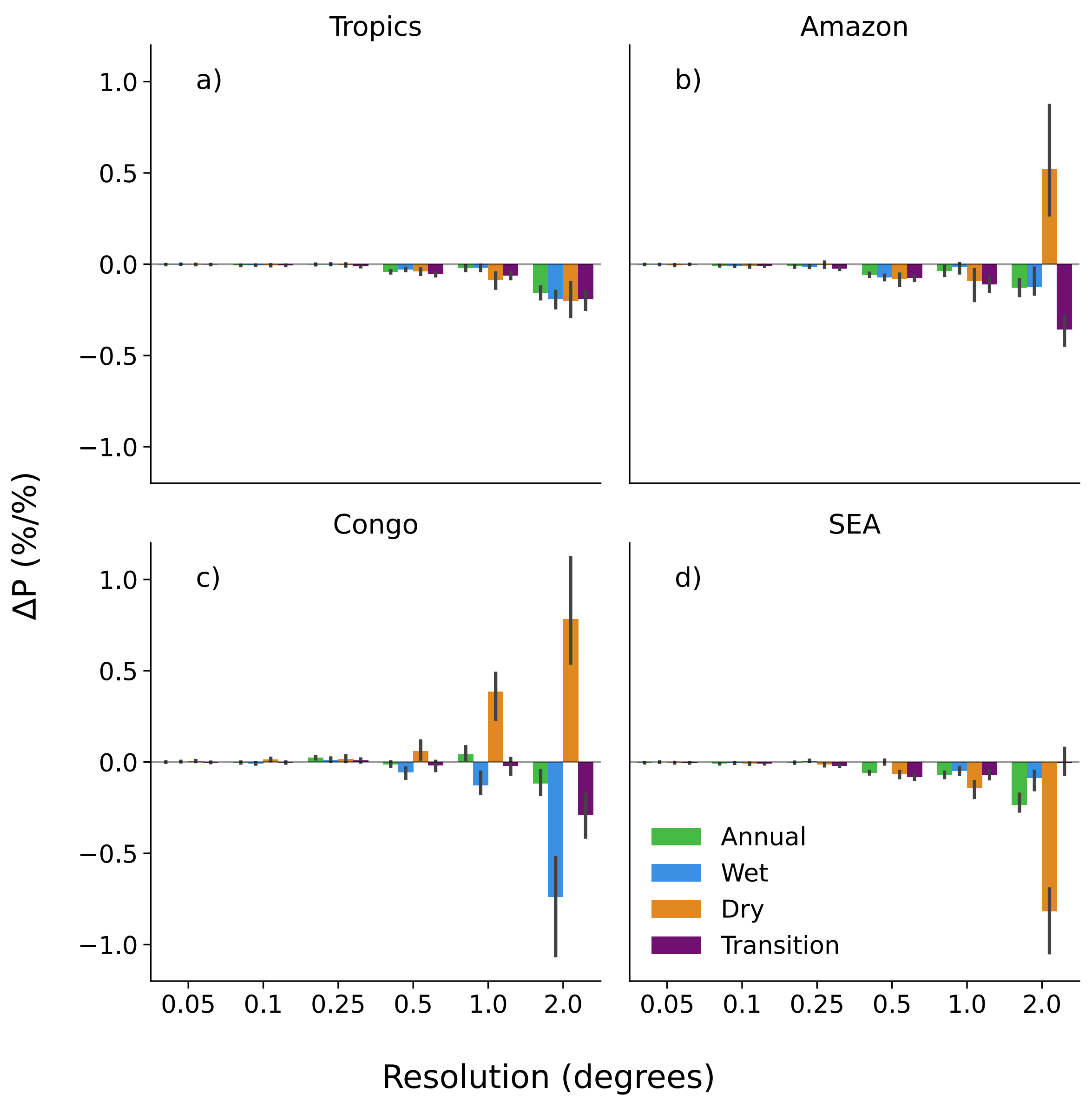

Supplementary Figure 2. Relative annual and seasonal changes in precipitation (P) due to forest cover loss. Bars show the median relative change in P per percentage point forest cover loss (%/%) for (a) Tropics, b) Amazon, c) Congo, d) SEA between 2003 - 2017. Results shown for forest loss scales of 0.05°, 0.1 0.25, 0.5°, 1.0°, 2.0° for satellite datasets. Error bar shows ± 1 standard error from the mean. Details of each data product are provided in Extended Data Table 1.

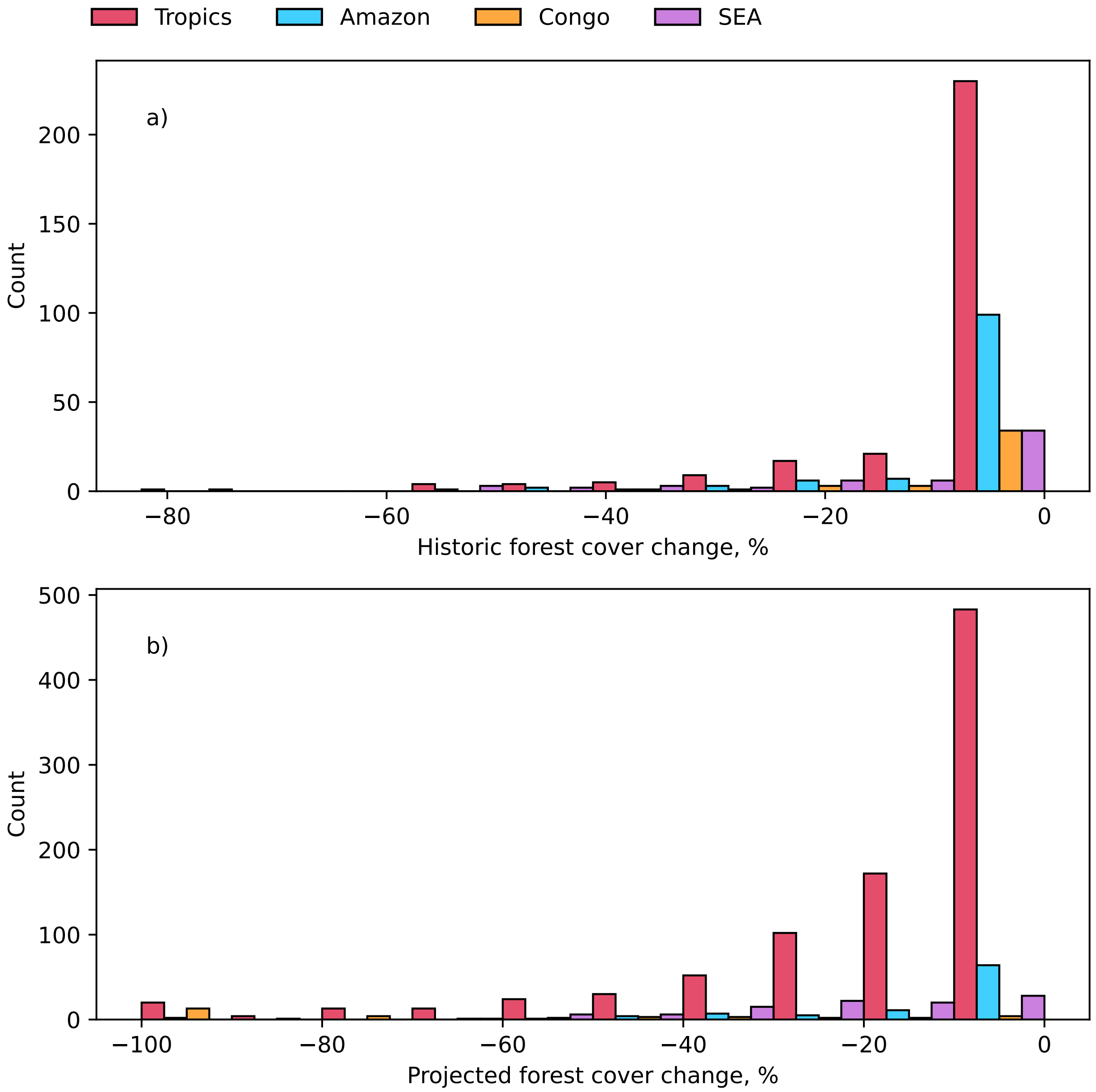

Supplementary Figure 3. Distribution of historic and projected change in forest cover (%). a) observed (2003-2017) from satellite and b) projected (2015-2100) from the GCAM model. Data analysed at 2° and coloured by region. Details of each data product are provided in Extended Data Table 1. Full results are available in an online repository [https://doi.org/10.5281/zenodo.7373832].

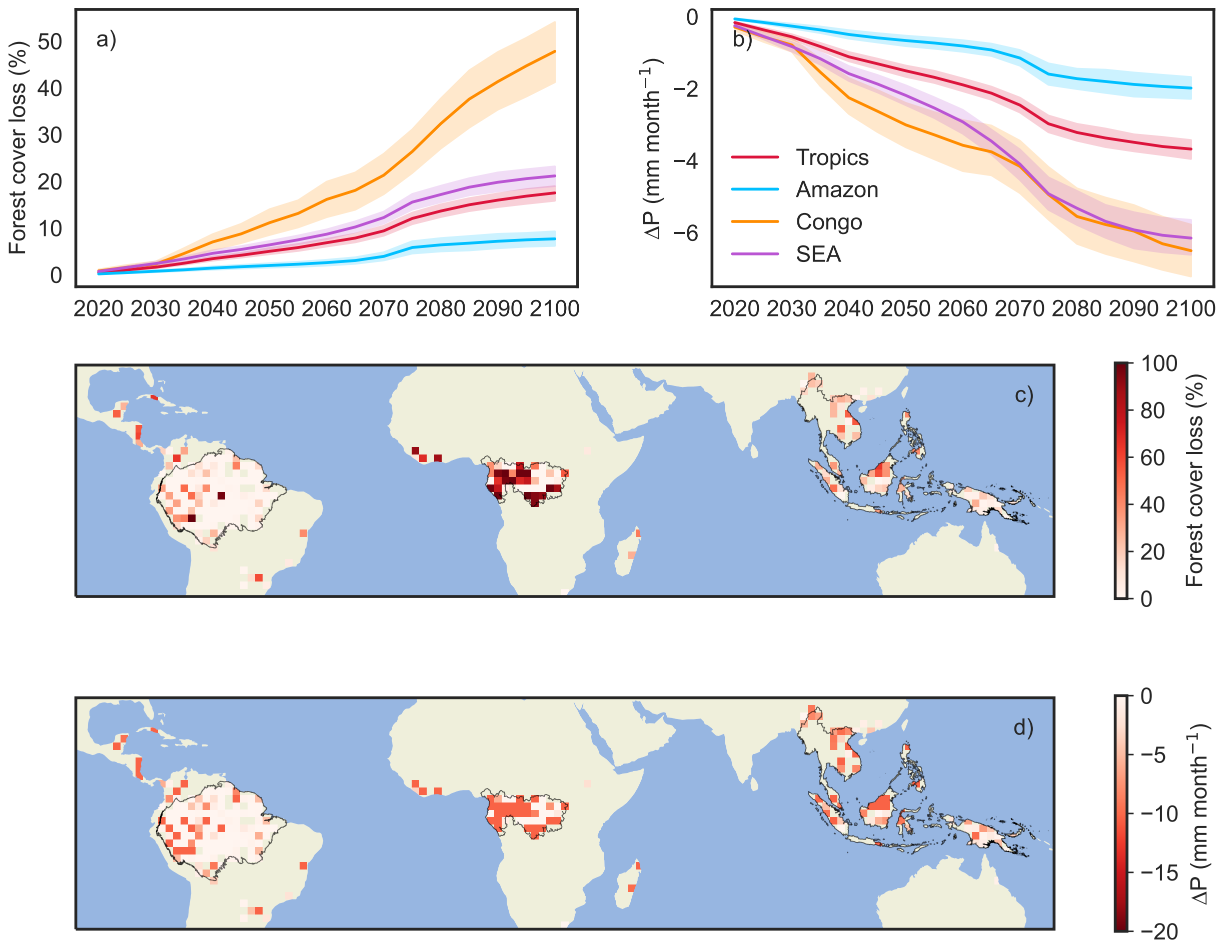

Supplementary Figure 4. Impact of projected future forest loss capped to 30%, on annual-mean precipitation. a) Mean forest cover loss over 2015 – 2100 under SSP3-4.5 for the tropics, Amazon, Congo and Southeast Asia (SEA); b) impact of projected forest cover loss on precipitation (P) (± 1 standard error from the mean); Spatial pattern of c) forest cover loss and; d) predicted P change (ΔP) in 2100 due to forest cover loss. Results are shown for 2.0° resolution. As Fig. 4 (main text) but impacts of forest loss on precipitation are capped at 30% forest loss (see methods). Maps of the different regions generated using Cartopy[73] and Natural Earth. Details of each data product are provided in Extended Data Table 1. Full results are available in an online repository [https://doi.org/10.5281/zenodo.7373832].

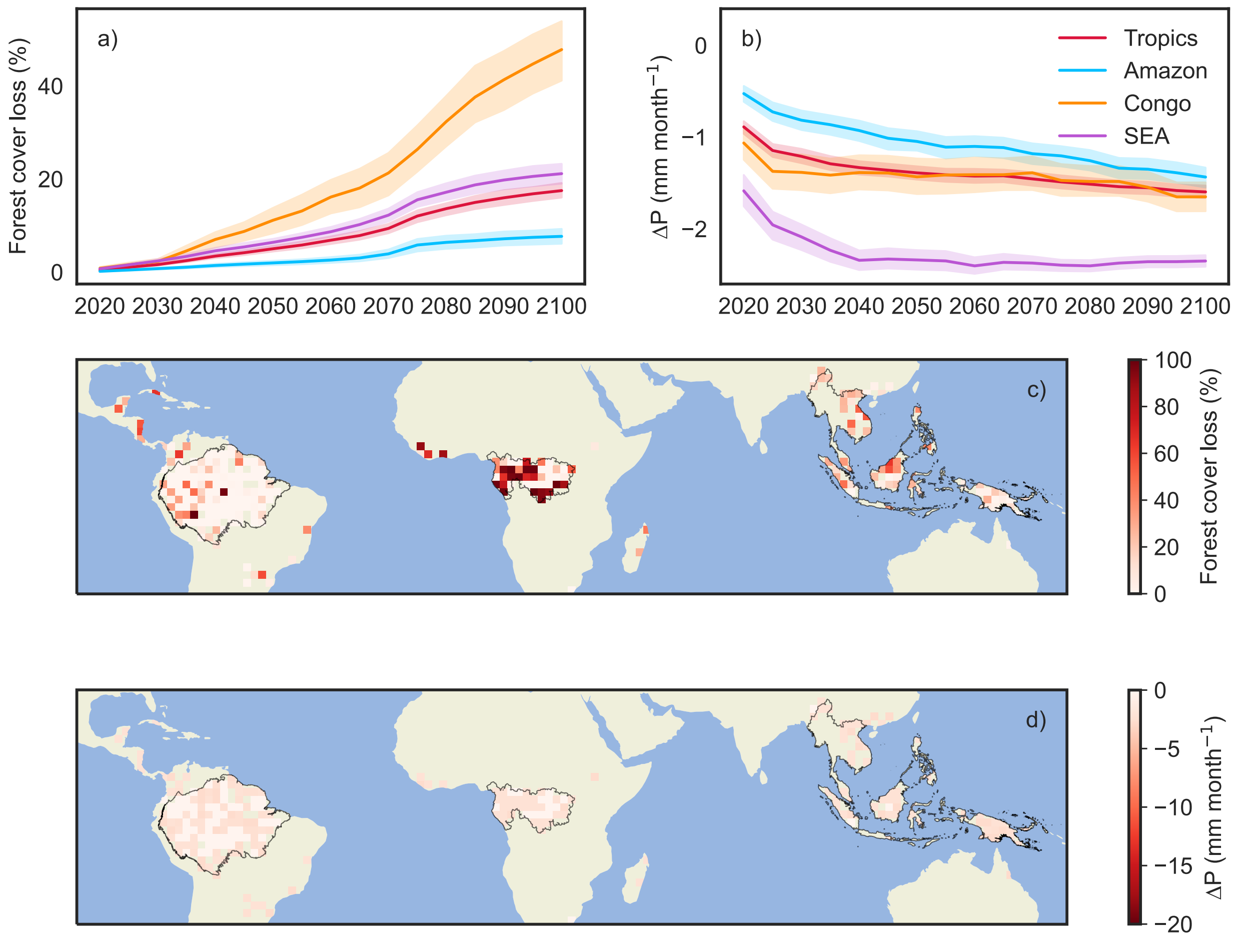

Supplementary Figure 5. Impact of projected future forest loss from 2015 to 2100 on annual-mean precipitation using a non-linear function. a) Mean forest cover loss under SSP3-4.5 for the tropics, Amazon, Congo and Southeast Asia (SEA); b) impact of projected forest cover loss on P (± 1 standard error from the mean); Spatial pattern of c) forest cover loss and; d) predicted P change (ΔP) in 2100 due to forest cover loss. As Fig. 4 (main text) but impacts of forest loss on precipitation are capped at 30% forest loss and a non-linear function based on Extended Data Fig. 1 is used to relate precipitation change to forest loss (see methods). Maps of the different regions generated using Cartopy[73] and Natural Earth. Details of each data product are provided in Extended Data Table 1. Full results are available in an online repository [https://doi.org/10.5281/zenodo.7373832].

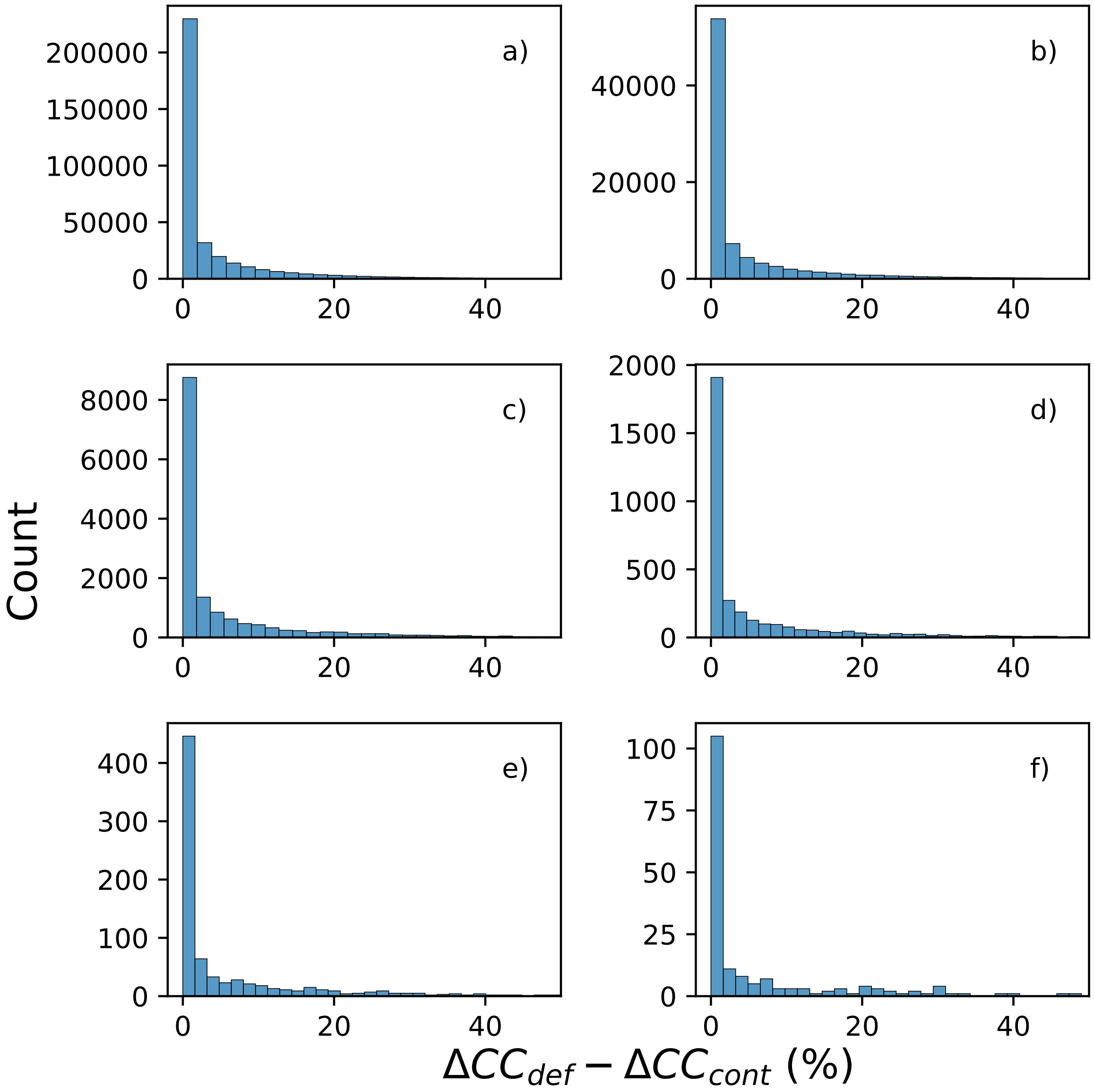

Supplementary Figure 6. Distribution of change in canopy cover (CC) for deforested pixels minus the change in canopy cover for control pixels, over the period 2003-2017. Results are shown at a) 0.05°, b), 0.1°, c) 0.25°, d) 0.5°, e) 1.0°, f) 2.0°. Details of each data product are provided in Extended Data Table 1. Full results are available in an online repository [https://doi.org/10.5281/zenodo.7373832].

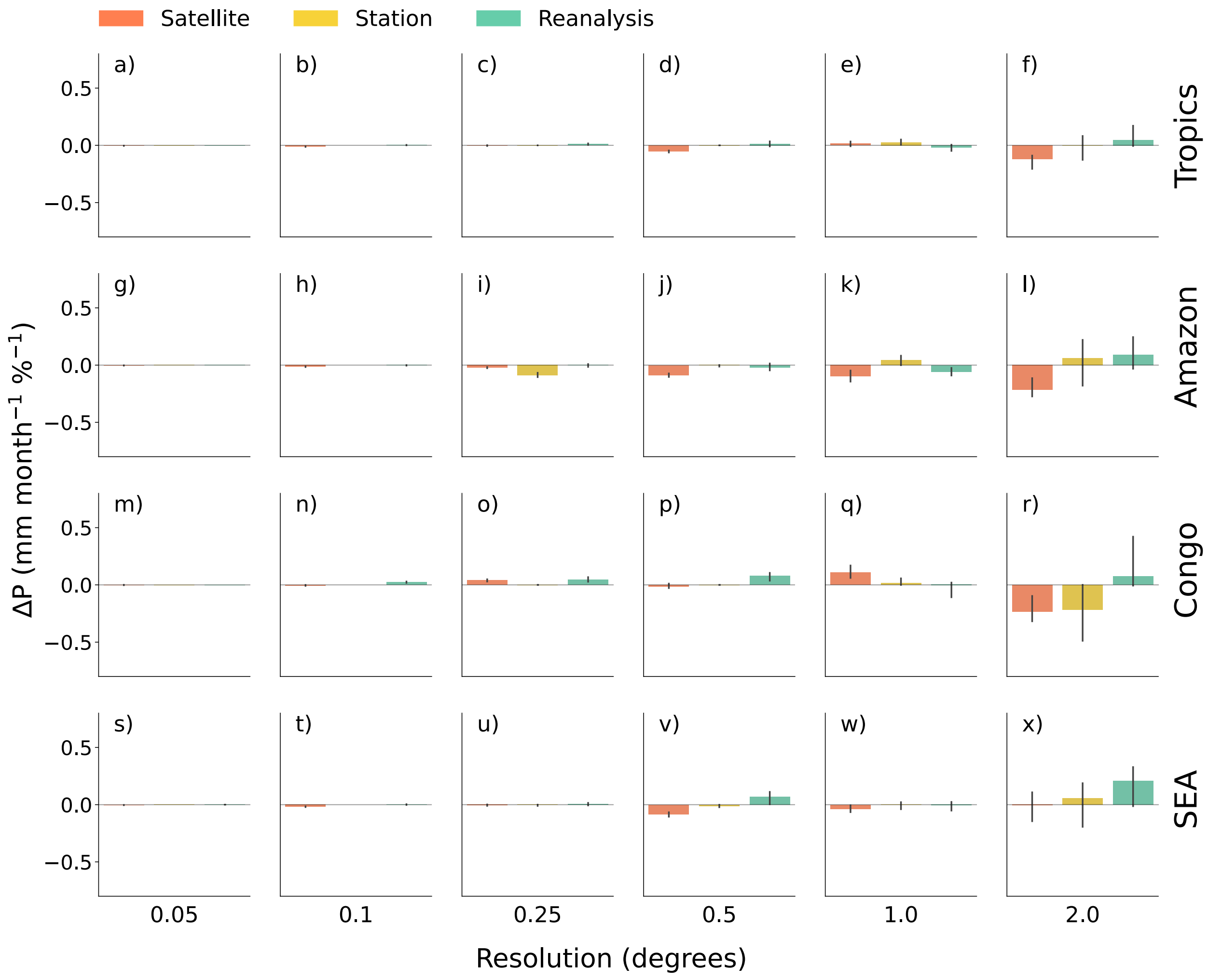

Supplementary Figure 7. Annual precipitation (P) change due to forest loss calculated with restrictions on background P. Results are shown for 5-year averages (2003-2007 & 2016-2020) with a 3x3 moving window and restricted to control and deforested pixels where the annual mean P differs by less than 10%. Bars show the median absolute change in annual P (mm month^{-1}) per percentage point tree cover loss in each region (Tropics (a-f), Amazon (g-l), Congo (m-r), Southeast Asia (SEA) (s-x)) and for each precipitation dataset category (satellite, station and reanalysis). Shown for forest loss scales of 0.05° (a, g, m, s), 0.1° (b, h, n ,t), 0.25° (c, i, o, u), 0.5° (d, j, p ,v), 1.0° (e, k, q, w), 2.0° (f, l , r, x). Error bars show ± 1 standard error from the mean. Details of each data product are provided in Extended Data Table 1. Full results are available in an online repository [https://doi.org/10.5281/zenodo.7373832].

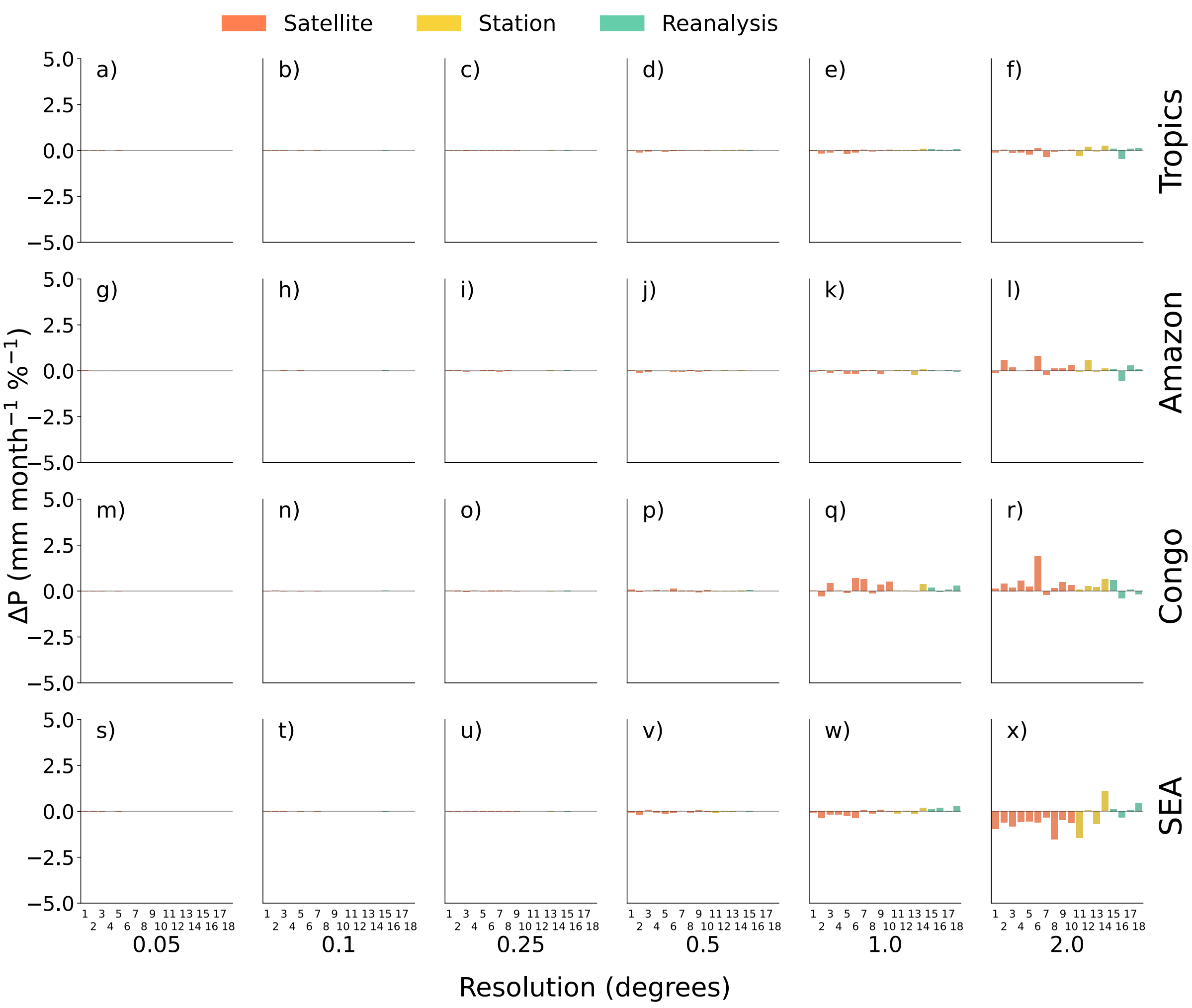

Supplementary Figure 8. Dry season precipitation (P) change due to forest loss during 2003 – 2017 for individual P datasets. Results are shown for 5 year averages and 3x3 moving window. Bars show the median absolute change in dry season P (mm month^{-1}) per percentage point forest cover loss in each region (Tropics, Amazon, Congo, SEA). Each P dataset is shown separately and ordered and coloured by category: satellite (orange), station (yellow) and reanalysis (turquoise). The datasets are numbered; 1) CHIRPS, 2) CMORPH, 3) CPC, 4) CRU, 5) ERA5, 6) GPCC, 7) GPCP, 8) GPM, 9) JRA, 10) MERRA-2, 11) NOAA 12) PERSIANN-CCS, 13) PERSIANN-CCSCDR, 14) PERSIANN-CDR, 15) PERSIANN-NOW, 16) PERSIANN, 17) TRMM, 18) UDEL. Results are shown for forest loss scales of 0.05°, 0.1°, 0.25°, 0.5°, 1.0°, 2.0°. Details of each data product are provided in Extended Data Table 1. Full results are available in an online repository [https://doi.org/10.5281/zenodo.7373832].

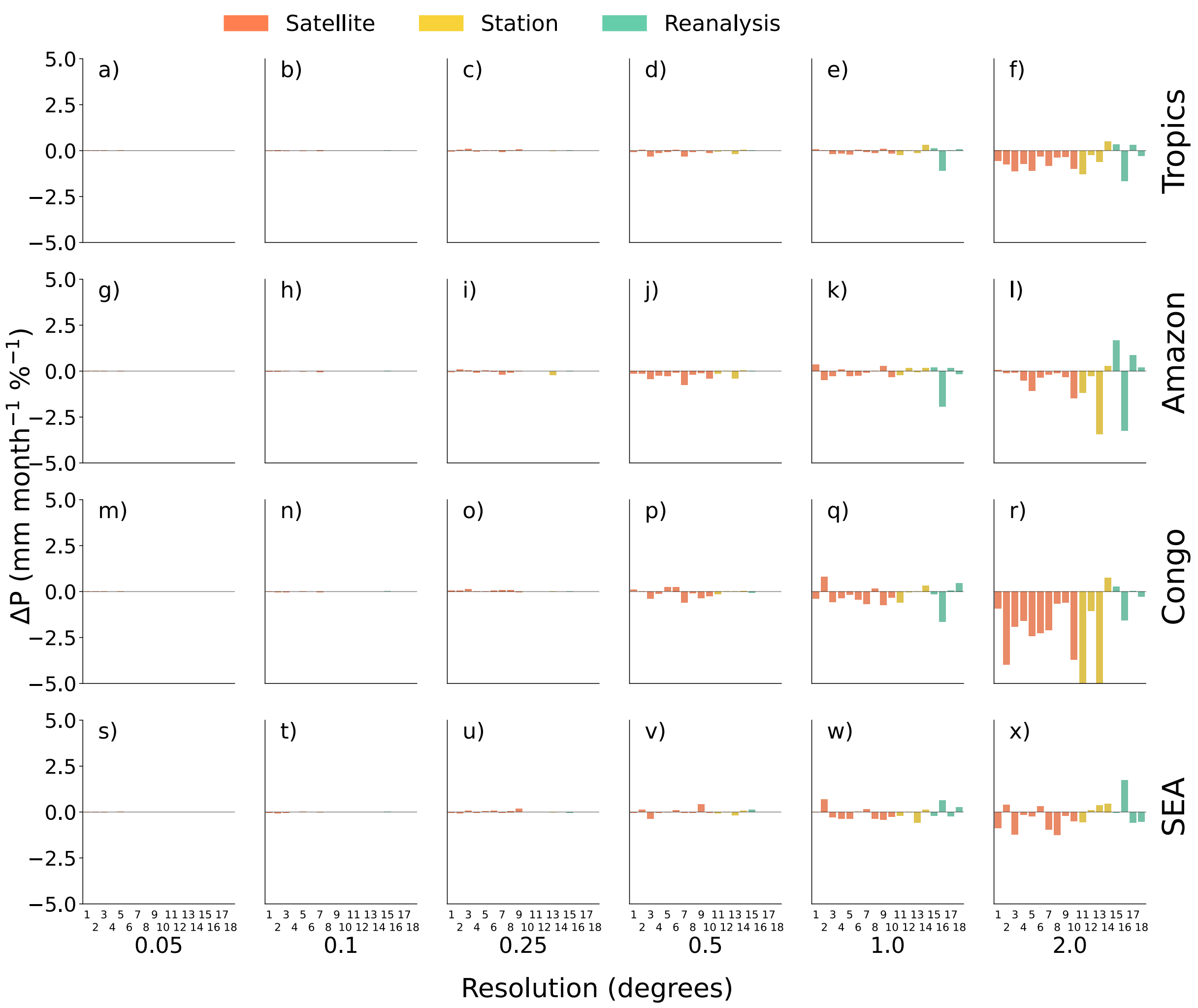

Supplementary Figure 9. Wet season precipitation (P) change due to forest loss during 2003 – 2017 for individual P datasets. Results are shown for 5-year averages and 3x3 moving window. Bars show the median absolute change in wet season P (mm month^{-1}) per percentage point forest cover loss in each region (Tropics, Amazon, Congo, SEA). Each P dataset is shown separately and ordered and coloured by category: satellite (orange), station (yellow) and reanalysis (turquoise). The datasets are numbered; 1) CHIRPS, 2) CMORPH, 3) CPC, 4) CRU, 5) ERA5, 6) GPCC, 7) GPCP, 8) GPM, 9) JRA, 10) MERRA-2, 11) NOAA 12) PERSIANN-CCS, 13) PERSIANN-CCSCDR, 14) PERSIANN-CDR, 15) PERSIANN-NOW, 16) PERSIANN, 17) TRMM, 18) UDEL. Results are shown for forest loss scales of 0.05°, 0.1°, 0.25°, 0.5°, 1.0°, 2.0°. Details of each data product are provided in Extended Data Table 1. Full results are available in an online repository [https://doi.org/10.5281/zenodo.7373832].

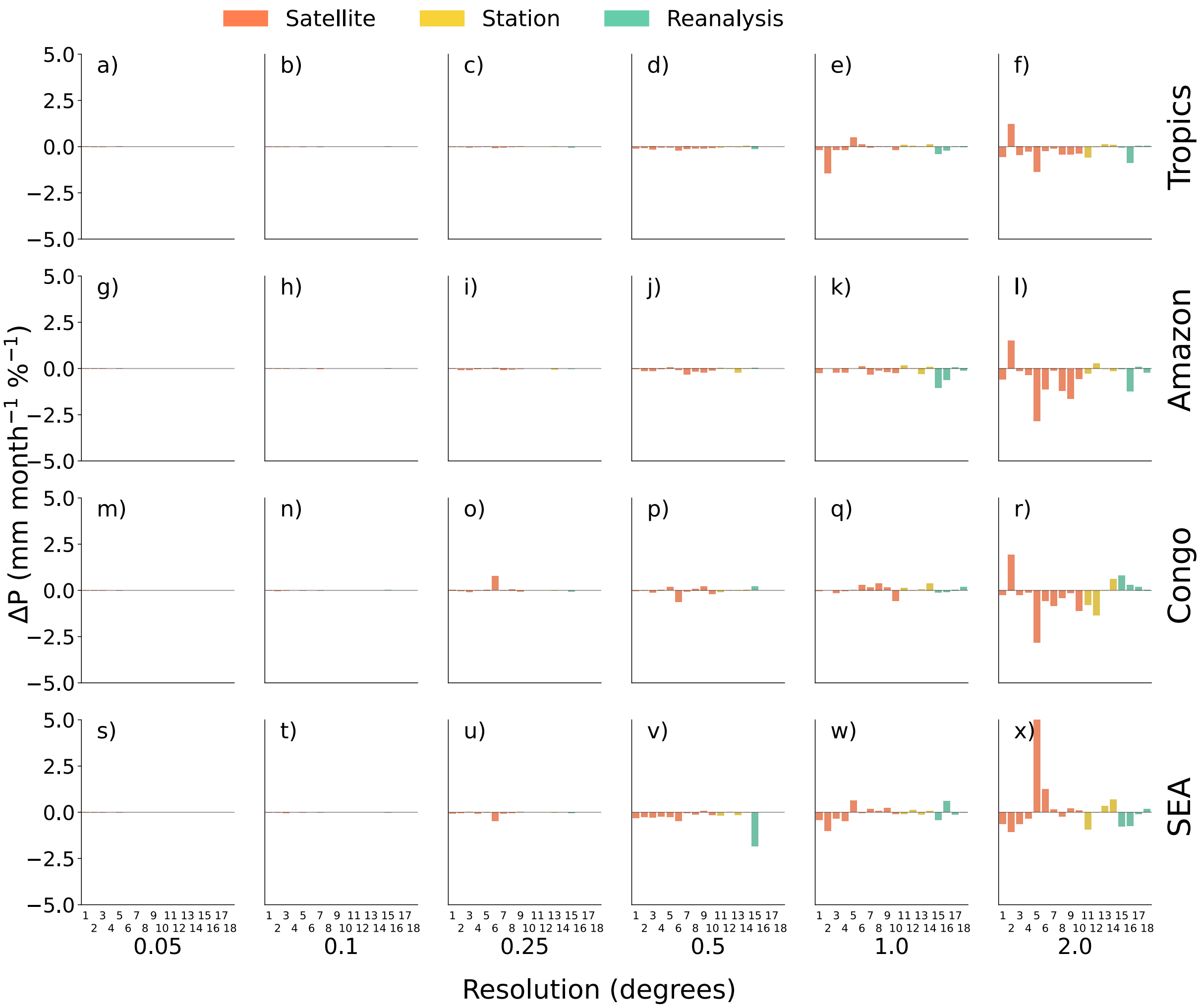

Supplementary Figure 10. Transition season precipitation (P) change due to forest loss during 2003 – 2017 for individual P datasets. Results are shown for 5-year averages and 3x3 moving window. Bars show the median absolute change in transition season P (mm month^{-1}) per percentage point forest cover loss in each region (Tropics, Amazon, Congo, SEA). Each P dataset is shown separately and ordered and coloured by category: satellite (orange), station (yellow) and reanalysis (turquoise). The datasets are numbered; 1) CHIRPS, 2) CMORPH, 3) CPC, 4) CRU, 5) ERA5, 6) GPCC, 7) GPCP, 8) GPM, 9) JRA, 10) MERRA-2, 11) NOAA 12) PERSIANN-CCS, 13) PERSIANN-CCSCDR, 14) PERSIANN-CDR, 15) PERSIANN-NOW, 16) PERSIANN, 17) TRMM, 18) UDEL. Results are shown for forest loss scales of 0.05°, 0.1°, 0.25°, 0.5°, 1.0°, 2.0°. Details of each data product are provided in Extended Data Table 1. Full results are available in an online repository [https://doi.org/10.5281/zenodo.7373832].
